# Intraocular Pressure Measurements in Standing Position with a Rebound Tonometer

**DOI:** 10.3390/medicina55100701

**Published:** 2019-10-18

**Authors:** Maddalena De Bernardo, Maria Borrelli, Giovanni Cembalo, Nicola Rosa

**Affiliations:** 1 Department of Medicine Surgery and Dentistry, University of Salerno, 84081 Baronissi, Italy; gcembalo@unisa.it (G.C.); nrosa@unisa.it (N.R.); 2 Department of Ophthalmology, Heinrich-Heine-University Düsseldorf, 40204 Duesseldorf, Germany; maria.borrelli@gmail.com

**Keywords:** intraocular pressure, Icare PRO, standing position, sitting position, supine position

## Abstract

*Background and Objectives:* It has been established that body position can play an important role in intraocular pressure (IOP) fluctuation. IOP has been previously shown to increase significantly when lying down, relative to sitting; this type of investigation has not been extensively reported for the standing (ST) position. Therefore, this study aims to look for eventual significant IOP changes while ST, sitting, and lying down. *Materials and Methods:* An Icare PRO was used to measure the IOP of 120 eyes of 60 healthy individuals, with age ranging from 21 to 55 years (mean 29.22 ± 9.12 years), in sitting, supine and ST positions; IOP was measured again, 5 min after standing (ST-5m). *Results:* Mean IOP difference between sitting and ST position was 0.39 ± 1.93 mmHg (95% CI: 0.04 to 0.74 mmHg) (*p* = 0.027); between sitting and ST-5m, it was −0.48 ± 1.79 mmHg (95% CI: −0.8 to −0.16 mmHg) (*p* = 0.004); between the sitting and supine position, it was −1.16±1.9 mmHg (95% CI: −1.5 to −0.82 mmHg) (*p* < 0.001); between the supine and ST position, it was 1.55 ± 2.04 mmHg (95% CI: 1.18 to 1.92 mmHg) (*p* < 0.001); between supine and ST-5m, it was 0.68 ± 1.87 mmHg (95% CI: 0.34 to 1.02 mmHg) (*p* < 0.001); and between ST-5m and ST, it was 0.94 ± 1.95 mmHg (95% CI: 0.58 to 1.29 mmHg) (*p* < 0.001). Mean axial eye length was 24.45 mm (95% CI: 24.22 to 24.69 mm), and mean central corneal thickness was 535.30 μm (95% CI: 529.44 to 541.19 μm). *Conclusion:* Increased IOP in the ST-5m position suggests that IOP measurements should be performed in this position too. The detection of higher IOP values in the ST-5m position than in the sitting one, may explain the presence of glaucoma damage or progression in apparently normal-tension or compensated patients.

## 1. Introduction

Intraocular pressure (IOP) measurement is an important part of the routine ophthalmic examination. Its elevation is a well-established risk factor for the development and progression of glaucoma [1,2,3]. Notably, despite improvements regarding the knowledge of glaucoma pathophysiology, IOP remains the only factor that can be modified by glaucoma therapy. IOP exhibits circadian fluctuation, and measurement at a single time could overlook temporary increases that may influence glaucoma progression [4,5,6]. Body position can play an important role in IOP fluctuation; indeed, IOP has been shown to considerably increase when a person is lying down, compared to the sitting position; this could partially explain some nocturnal elevations [7,8,9]. In some situations, this could contribute to continued glaucomatous damage, despite the observance of low IOP during routine ophthalmic examinations; thus, such patients have been advised to sleep with their heads elevated [10].

We suspected that significant changes in IOP might occur in the standing (ST) position. In fact, to the best of our knowledge, the IOP in the ST position was only studied in two small groups of healthy subjects and multiple system atrophy patients where only 11 subjects were evaluated [11].

Therefore, we investigated whether such IOP changes occur in a normal population. The gold standard for measuring IOP is through Goldmann applanation tonometry (GAT). However, GAT cannot be used when the patient is in a supine or ST position; thus, this study was performed using an Icare PRO (ICP).

## 2. Materials and Methods

One hundred and twenty eyes of 60 patients (14 men), with an age range of 21 to 55 years (mean 29.22 ± 9.12 years) and a refractive error range of −6.63 to +1 (mean −2.38 ± 2.57), were included in this prospective observational study. The study was conducted in accordance with the tenets of the World Medical Association’s Declaration of Helsinki, and approval was obtained from our Institutional Review Board (Approval number n.33, approved on 10.03.2017). Informed consent for this research was obtained from the patients. Each subject underwent a general physical checkup to confirm the absence of any systemic disease that could interfere with the present study. Participants underwent a comprehensive ophthalmic examination that included best-corrected visual acuity, refractive error, a fundus examination, an axial eye length (AL) evaluation with an IOLMaster (Zeiss, Jena, Germany, version 5.4.4.00006), and a central corneal thickness (CCT) measurement with a Pentacam Hr (Oculus, Wetzlar, Germany, version 1.19r11).

IOP was measured between 10:00 and 12:00 by a single observer with an ICP (Icare Finland Oy, Finland version 1.1) that utilized an impact rebound technique: a small probe is accelerated against the cornea and the rebound acceleration is measured, then converted to IOP [12]. This technique does not require calibration or local anesthesia because the contact with the corneal surface is very brief. The ICP automatically averages six consecutive IOP measurements and provides the mean IOP of these six measurements; the reliability of the measurements is indicated by a color code displayed below the IOP result. The indicator is green if the variation is within normal limits and yellow when the variation is above normal; the measurements should be viewed with caution when the variation is unacceptably high (i.e., the indicator is red) [13]. 

IOP measurements were obtained in the sitting position, in the supine position after 5 min of lying down, in the ST position, and after 5 min in the ST position (i.e., ST-5m), as this methodology was used by Singleton et al. [11].

Statistical evaluation was performed with a paired Student’s *t*-test utilizing an excel spreadsheet. Sample size was determined by maximizing the statistical power. The analysis was performed by using G*Power3.1 software [14]. A difference between the two dependent means (matched pairs) *t*-test was computed. Input data were the following: α was set at 0.01, 1−β was set at 0.99 and effect size was set as medium at around 0.4. Results were as follows: non-centrality parameter δ = 4.710; Critical t = 2.358; DF = 119; Actual power = 0.990095 and Total sample size = 120.

## 3. Results

**Figure 1:**
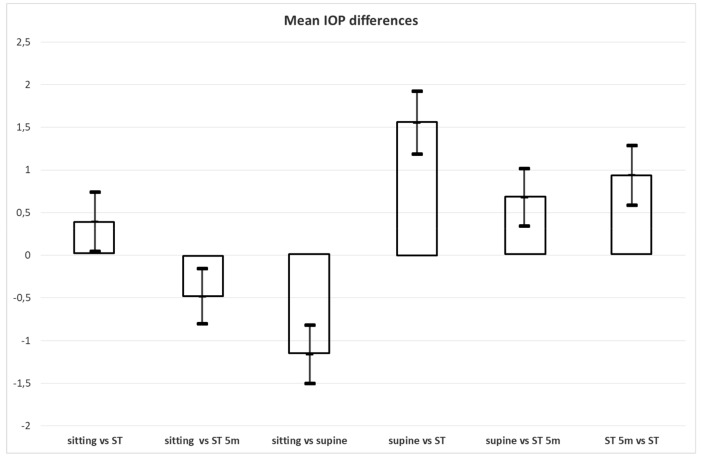
Differences in intraocular pressure (IOP) measurement in sitting, supine, standing (ST) and 5 min after standing (ST-5m) positions (in mmHg). Values are means and 95% CI.

**Figure 2:**
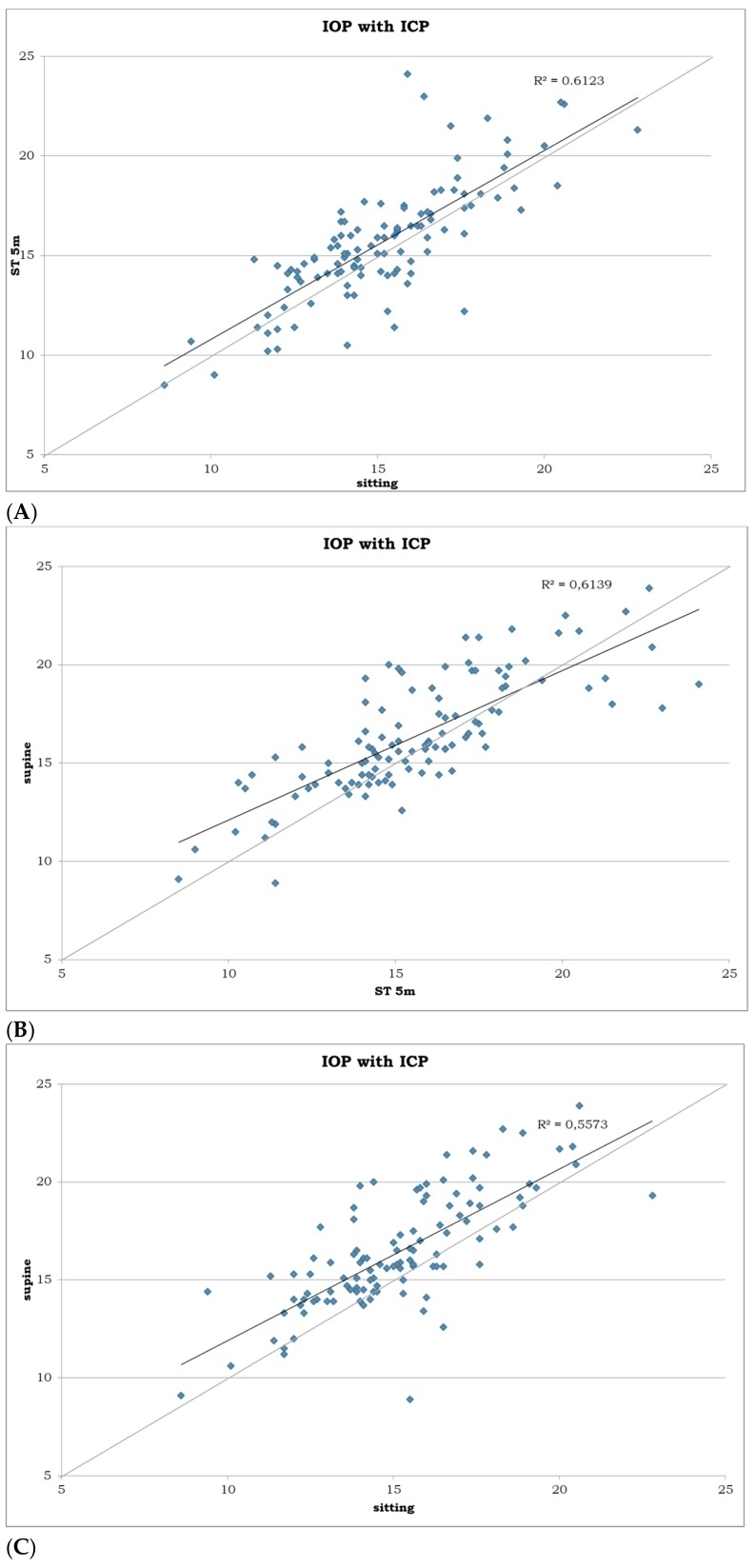

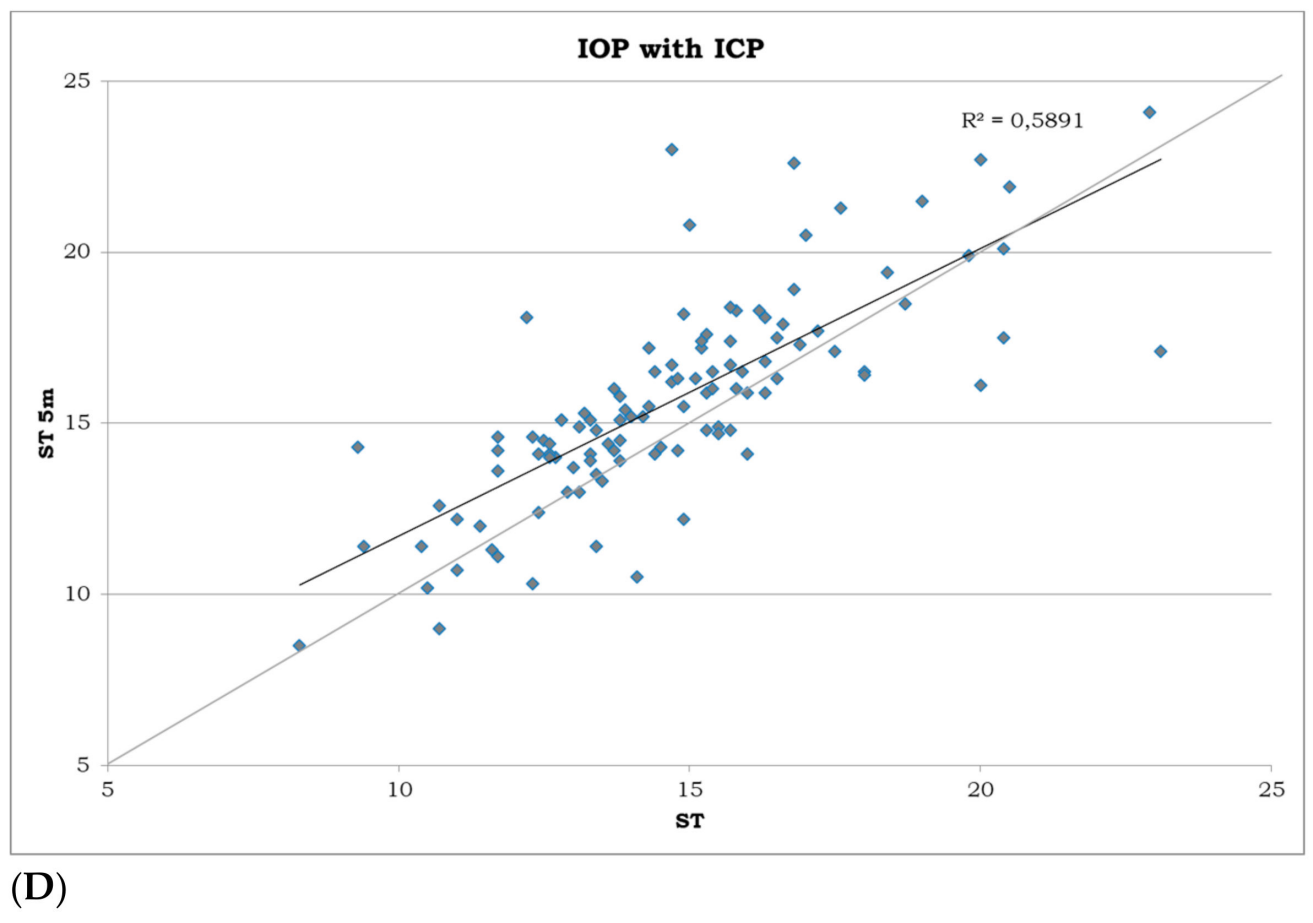
Scatterplots showing correlation between the IOP measurements (**A**) in sitting position on the horizontal axis and IOP measurement in ST-5m position on vertical axis (in mmHg); (**B**) in ST-5m position on the horizontal axis and IOP measurement in supine position on vertical axis (in mmHg); (**C**) in sitting position on the horizontal axis and IOP measurement in supine position on vertical axis (in mmHg); (**D**) and in ST position on the horizontal axis and IOP measurement in ST-5m position on vertical axis (in mmHg). R2 is a measure of goodness of fit of linear regression.

IOP in the sitting position ranged from 8.6 to 22.8 mmHg (mean, 15.13 mmHg; 95% CI, 14.68–15.58 mmHg); in the supine position, it ranged from 8.9 to 23.9 mmHg (mean, 16.4 mmHg; 95% CI, 15.87–16.92 mmHg); in the ST position, it ranged from 8.3 to 23.1 mmHg (mean, 14.7 mmHg; 95% CI, 14.21–15.19 mmHg); and, in the ST-5m, it ranged from 8.5 to 24.1 mmHg (mean, 15.65 mmHg; 95% CI, 15.12–16.19 mmHg). Differences in IOP measurement among the positions are summarized in Table 1 and Figure 1 and Figure 2A–D, where the sitting position was utilized as a reference position and the other values were subtracted from this position to better show the increase or decrease from the sitting one. The same criteria were used in the comparison between the supine and standing position, and between the two standing positions.

**Figure 3:**
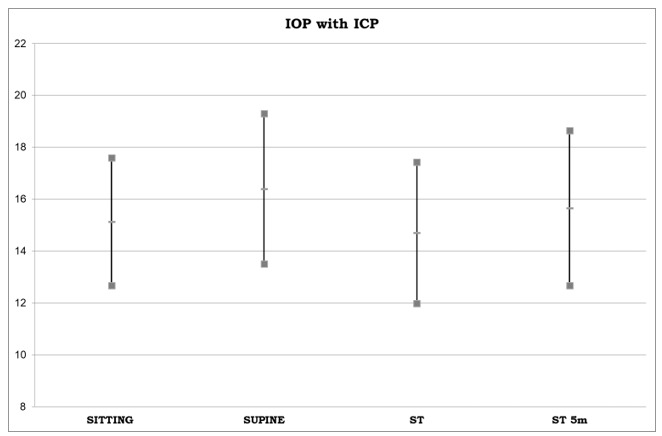
IOP measurements in sitting, supine, ST and ST content-type="color:#231F20">-5m positions. Values are means and standard deviations.

According to Table 1, there was a non-significant decrease in IOP from the sitting position to the ST position (mean ΔIOP = 0.39, *p* = 0.027) (first column); while in the ST-5m position, there was a small but significant increase in IOP compared to the sitting position (mean ΔIOP = −0.48, *p* = 0.004) (second column); in the supine position, IOP was higher than in the sitting position (mean ΔIOP = −1.16, *p* < 0.001)(third column); in the supine position, IOP was higher than in the ST position (mean ΔIOP = 1.55, *p* < 0.001) (fourth column), but this difference was reduced over time, as shown in the fifth column (mean ΔIOP = 0.68, *p* < 0.001), and in the sixth column (mean ΔIOP = 0.94, *p* < 0.001). Means and standard deviations for IOP measurement in the sitting, supine, ST, and ST-5m positions are illustrated in Figure 3.

**Figure 4:**
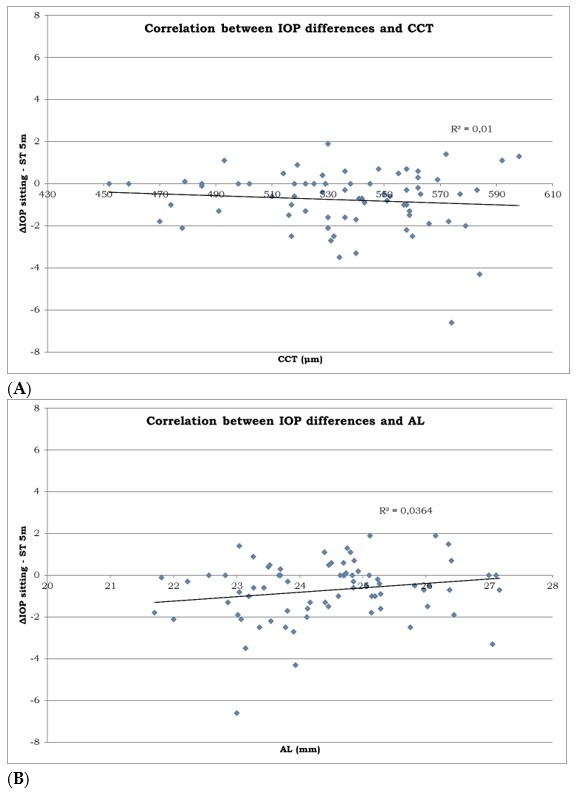
Scatterplots showing correlation between the IOP measurements (**A**) between the CCT on the horizontal axis (in microns) and difference in IOP measurements between the sitting and ST-5m position on the vertical axis (in mmHg). (**B**) between the AL on the horizontal axis (in millimeters) and difference in IOP measurement between sitting and ST-5m position on the vertical axis (in mmHg). *R*^2^ is a measure of goodness of fit of linear regression.

The mean CCT was 535.30 μm (95% CI: 529.44 to 541.19 μm). The mean AL was 24.45 mm (95% CI: 24.22 to 24.69 mm). There was no relationship between CCT and the difference in IOP between sitting and ST-5m positions (Figure 4A), nor between AL and the difference in IOP in the same positions (Figure 4B).

## 4. Discussion

In the international literature, we were able to find IOP measurements in the ST position in only two studies; neither used the ICP [11,15]. LeMarr et al. [15] evaluated changes in IOP in 26 young subjects, in the ST position compared with a very unusual position (head down); they found that IOP increased in the head-down position, but did not perform a comparison with sitting or supine positions. In the other study, Singleton et al. [11] compared postural changes in IOP in only 11 normal subjects, relative to several groups of patients who had different types of autonomic dysfunction; they concluded that no statistically significant postural changes in IOP occurred in normal subjects. Additionally, multiple authors reported measuring IOP in sitting and supine positions using different devices, such as the ICP [16], TonoPen [17,18,19,20,21], and AVIA [13]. Some others compared different tonometers [13,22], but we found no reports of IOP measurements in subjects in the ST position.

The ICP tonometer was used to measure IOP in 40 eyes of 20 healthy Korean subjects; the investigators reported a statistically significant increase in mean IOP (*p* < 0.001) of roughly 2 mmHg upon postural change from the sitting to the supine position [16]. The Tono Pen has been used more frequently. In one study, IOP was measured in 19 healthy subjects; first while sitting, then in the supine position after lying down for 15 ± 5 min and for 45 ± 5 min; the authors reported a mean increase of 1.6 mmHg after 15 min in the supine position, which decreased to 1.1 mmHg after 45 min in the supine position, with no statistically significant differences between measurements at 15 and 45 min [17]. In another study, IOP was measured in 19 healthy young Korean subjects in the sitting and supine positions, revealing an increase of approximately 3 mmHg from the first to the second position [19]. In another investigation, the same device was used to measure IOP in both sitting and supine positions in 45 glaucoma patients and 46 healthy subjects; both groups showed a statistically significant increase in mean IOP from the sitting position to supine position, which was higher in glaucomatous patients (2.3 mmHg) than in healthy subjects (1.2 mmHg) [21]. 

In a comparison between an ICP and a TonoPen AVIA, Schweier et al. measured the IOP in 36 eyes of 36 healthy individuals—first in the sitting position, then 10 min after reclining. They found that IOP was lower in the sitting position than in the reclining position with both hand-held tonometers; the mean difference was greater with a TonoPen AVIA (1.8 mmHg) than with an ICP (0.8 mmHg) [13]. Furthermore, they evaluated the coefficient of variation (COV) of the different instruments used, finding that ICP had a COV equal to the TonoPen AVIA one in sitting position, 0.052, whereas the COV for GAT was 0.029 [13].

In another comparison between Tonopen XL and ICP, Barkana et al. measured IOP in 21 eyes of 21 healthy subjects in the sitting position, then after lying in the supine position for 10 min; they found a mean increase in IOP of 0.9 mmHg from the sitting position to the supine position with the TonoPen. In contrast, the ICP showed a mean reduction of 0.9 mmHg from the sitting position to the supine position [22]. 

Our results, obtained with ICP, confirm the findings by Lee et al. [16] and Schweier et al. [13], but contrast with those obtained by Barkana [22] because we found an increase in mean IOP upon positional change from the sitting to supine position. However, we suspect that the finding of a significant increase in IOP upon prolonged duration in the ST position may be more clinically meaningful. An increase in IOP in the supine position may have a variety of explanations; it has been suggested that this may occur as a result of choroidal vascular engorgement caused by the redistribution of body fluids in the supine position [23,24], or an increase in episcleral venous pressure in the supine position [25,26,27]. In contrast, the potential reason for increased IOP upon a prolonged duration in the ST position is unclear. Although the exact mechanism of IOP regulation is unknown, it is suspected to be under systemic vascular control [11]. Indeed, the autonomous nervous system, which involves local myogenic metabolic and circulating humoral agents, contributes to blood flow control throughout the vital organs via autoregulation; thus, it maintains relatively constant blood flow to tissues, despite fluctuations in perfusion pressure [11,28,29]. 

One potential weakness of the present study is that we used the ICP, rather than the GAT, which represents the gold standard for measuring IOP [30]. Regrettably, several corneal parameters, such as biomechanical properties [31,32], CCT [33,34,35], corneal astigmatism [36,37], corneal irregularities [38], and a history of refractive corneal surgery can influence IOP measurements acquired by GAT. Moreover, the purpose of the study was to check the differences between the sitting position and the other positions; importantly, the GAT cannot be used in the ST or supine positions, or if a patient is unable to sit at a slit lamp (e.g., bedridden persons and small children). Thus, we selected the ICP as a hand-held device for IOP measurement.

Another potential weakness is that we examined only healthy subjects, because there is no evidence in the literature about these kind of measures. In these subjects, we found a significant difference in IOP both between the sitting position compared to the ST-5m one, and between the ST and the ST-5m. Although we are aware that these IOP changes are small, clinicians should be aware of these fluctuations, as they could have the same importance in inducing ocular hypertension as, for instance, when the patients are lying supine.

## 5. Conclusions

In conclusion, our results suggest that IOP measurement should be performed not only in the sitting and supine positions, it should also be performed after 5 min in the ST position. Importantly, some types of jobs (e.g., waiters, policemen, and chefs) involve a considerable amount of time in an ST position, which may explain glaucoma progression in some apparently compensated patients.

## Figures and Tables

**Table 1:** Intraocular pressure difference (in mmHg) measured with Icare Pro tonometer between different positions.

	Difference between Sitting and ST^‡^	Difference between Sitting and ST-5m^†^	Difference between Sitting and Supine	Difference between Supine and ST	Difference between Supine and ST-5m	Difference between ST-5m and ST
Mean	0.39	−0.48	−1.16	1.55	0.68	0.94
SD	1.93	1.79	1.90	2.04	1.87	1.95
Min.	−7	−8.20	-5.80	−3.90	−5.20	−6
Max.	5.90	5.40	6.60	7.10	5.20	8.30
*p*	0.027	0.004	<0.001	<0.001	<0.001	<0.001
95% CI	0.04 to 0.74	−0.8 to −0.16	−1.5 to −0.82	1.18 to 1.92	0.34 to 1.02	0.58 to 1.29

† measurement obtained after five minutes in standing position; ‡ measurement obtained immediately after assuming standing position
